# Life cycle of an n-globin pseudogene microsatellite locus

**DOI:** 10.3389/fgene.2013.00267

**Published:** 2013-12-04

**Authors:** Jasmin H. Bavarva, Hongseok Tae, Pawel Michalak, Harold R. Garner

**Affiliations:** Virginia Bioinformatics Institute, Virginia Polytechnic Institute and State UniversityBlacksburg, VA, USA

**Keywords:** microsatellites, n-globin, evolution, microsatellite life cycle, gorilla

Microsatellites are composed of tandemly repeated short motifs of 1–6 nucleotides. They are common in eukaryotic genomes; in humans they make up as much as 3% of the genome (Lander et al., 2001). These repeat-containing loci tend to be hypervariable with variation occurring among individuals of the same species as well as between species. The origin and evolution of microsatellites remain a major puzzle. The birth of a tetranucleotide repeat (ATGT) in the lineage leading to African apes (gorilla, bonobo and chimpanzee) and humans was documented in the n-globin pseudogene (Messier et al., 1996). To test whether the locus is under further expansion or degeneration or if there are any variations as proposed in the life-cycle of the microsatellites (Buschiazzo and Gemmell, 2006), we analyzed positions chr11:5263801:5263831 (hg19) in 82 samples from the 1000 Genomes Project (Genomes Project et al., 2010). To obtain reliable read coverage for the locus, we selected Illumina exome sequencing data and visually inspected the INDEL length in the reads aligned to the locus. We found a repeat deletion (4 bases) of the 4-mer microsatellite locus in 46 samples (56%) relative to reference allele, as supported by at least two reads in each sample. We assume that the longer (reference) microsatellite allele is ancestral, as at least two mutations are reported in this allele (rs34312249 and rs147740082), suggesting a degeneration process of the pure microsatellite locus. The short allele had 28% frequency in analyzed samples.

**Figure 1:**
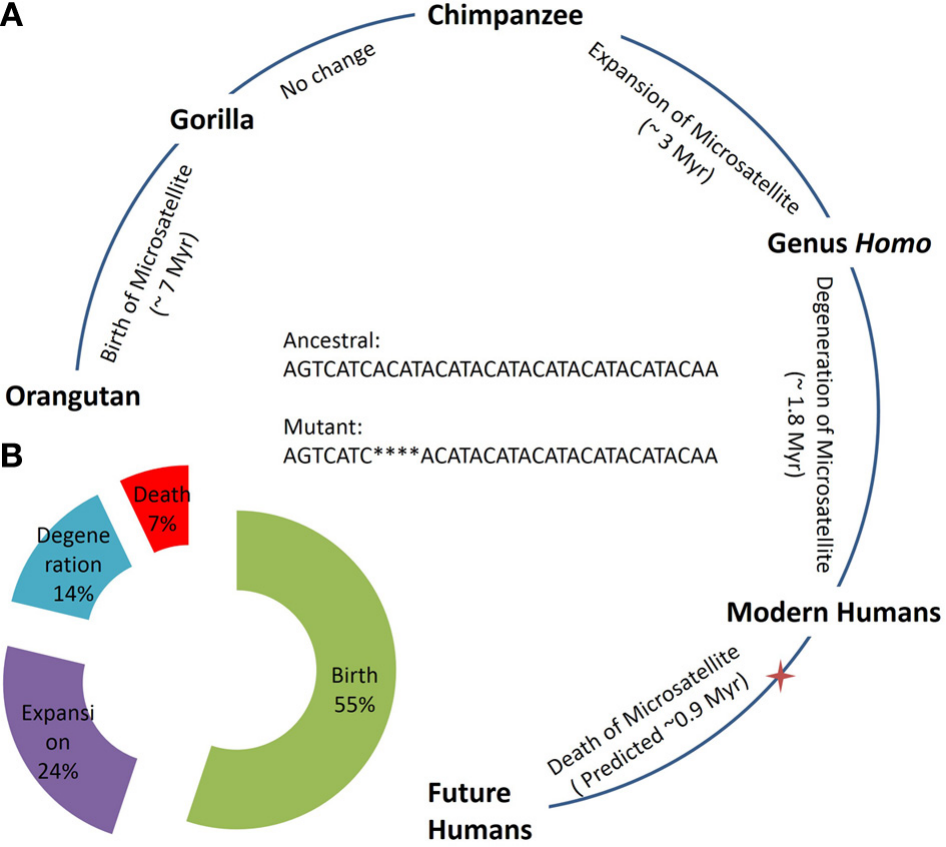
**Evolution and life cycle of an n-globin pseudogene microsatellite locus. (A)** Evolutionary time estimate for the species emergence and corresponding change in the n-globin pseudogene microsatellite locus, illustrating different stages of life cycle and predicting future steps. **(B)** Total percentage of time required for each stage in life cycle of n-globin pseudogene microsatellite locus as based on complete predicted life cycle in **Panel A**. Red star indicates the present humans on the evolution time scale and, that corresponds to the ~0.2 Myr. Hg19 genomic coordinates for the sequence: Chr11:5263798:5263832, positive strand.

The genus *Homo* is believed to have evolved 2.0 Myr (Curnoe, 2010) and modern humans 0.2 Myr (Vigilant et al., 1991) ago. Last evolutionary change in the n-globin pseudogene locus is documented between chimpanzees and humans when the microsatellite locus was expanded in humans. Unfortunately, intraspecies variation in the locus is unknown in chimpanzees and gorillas and we were unable to find the locus in the Neanderthal genome, presumably due to its incompleteness (Green et al., 2006). The locus alteration from orangutan to gorilla took ~7.0 Myr followed by species evolution with no change in the locus (gorilla to chimpanzee). Similarly, the evolution of the chimpanzee to the genus *Homo* took ~3.0 Myr with an accompanying n-globin microsatellite locus expansion. As time required for subsequent stage of life cycle seems to decrease by about 50% (Birth: ~7 Myr, Expansion: ~3 Myr, Degeneration: 1.8 Myr) (Figure 1A), it appears that the plausible death of the n-globin pseudogene microsatellite locus is expected within the next ~0.9 Myr (Figure 1B). However, human demography and unknown fitness effects of the microsatellite polymorphism can influence the locus evolutionary dynamics.

To test whether the scenario (life cycle of microsatellites) can be applied to other microsatellite loci, we reanalyzed our microsatellite data from multiple species (Galindo et al., 2009). We observed the birth, expansion, and degeneration of several microsatellite loci in four microsatellite motif families in human, chimpanzee, orangutan, rhesus macaque, marmoset and mouse genomes. Although, there were few loci that showed deviations from the expected life-cycle, such as AATGG repeat in *DMD* and *HEPH* gene that showed stability for an extended period of time, and in *PCNXL2* where it showed one repeat diminution in chimpanzee compared to orangutan and human, majority of the repeats arguably followed the proposed life cycle.

Overall, it would be of great scientific significance to constantly monitor a number of marker microsatellite loci for genetic deviations as time progresses and more samples are being sequenced.
